# High triglyceride/HDL cholesterol ratio is associated with silent brain infarcts in a healthy population

**DOI:** 10.1186/s12883-019-1373-8

**Published:** 2019-07-02

**Authors:** Ki-Woong Nam, Hyung-Min Kwon, Han-Yeong Jeong, Jin-Ho Park, Hyuktae Kwon, Su-Min Jeong

**Affiliations:** 1 Department of Neurology, Seoul National University College of Medicine and Seoul National University Hospital, Seoul, South Korea; 2 Department of Family Medicine, Seoul National University College of Medicine and Seoul National University Hospital, 101 Daehakno, Jongno-Gu, Seoul, 03080 South Korea; 3 Department of Neurology, Seoul National University College of Medicine and Seoul Metropolitan Government-Seoul National University Boramae Medical Center, Seoul, South Korea

**Keywords:** Triglycerides; High-density lipoprotein; Silent brain infarct; Lacune; Cerebral small vessel diseases; Metabolic syndrome

## Abstract

**Background:**

Triglycerides (TG)/high-density lipoprotein (HDL) cholesterol ratio is a marker of small/dense low-density lipoprotein particles, which are closely associated with various metabolic and vascular diseases. However, the role of TG/HDL cholesterol ratio in cerebrovascular diseases has not been well studied. In this study, we evaluated the relationship between TG/HDL cholesterol ratio and the presence of silent brain infarct (SBI) in a neurologically healthy population.

**Methods:**

We retrospectively evaluated consecutive participants in health check-ups between January 2006 and December 2013. SBI was defined as an asymptomatic, well-defined lesion with a diameter of ≥3 mm on T1- or T2-weighted images. TG/HDL cholesterol ratio was calculated after dividing absolute TG levels by absolute HDL cholesterol levels.

**Results:**

Of 3172 healthy participants, 263 (8.3%) had SBI lesions. In multivariate analysis, TG/HDL cholesterol ratio was independently associated with SBI (adjusted odds ratio [aOR] = 1.16, 95% confidence interval [CI] = 1.00 to 1.34, *P* = 0.047). This association was prominent in males (aOR = 1.23, 95% CI = 1.03 to 1.48, *P* = 0.021), but not in females. In the analyses of the relationships between lipid parameters and SBI lesion burden, TG/HDL cholesterol ratio was positively correlated, and total cholesterol/TG ratio was negatively correlated with SBI lesion burden, in dose-response manners (*P* for trend = 0.015 and 0.002, respectively).

**Conclusions:**

The TG/HDL cholesterol ratio was positively associated with the prevalence of SBI in a neurologically healthy population.

**Electronic supplementary material:**

The online version of this article (10.1186/s12883-019-1373-8) contains supplementary material, which is available to authorized users.

## Background

Silent brain infarct (SBI) is a subclinical pathology that is commonly found prior to ischemic stroke, especially in the elderly [1, 2]. Ischemic stroke often results in disabilities with limited neurological recoveries [3]. Therefore, it is important to evaluate modifiable risk factors for SBI and establish an early intervention prior to ischemic stroke. Several risk factors and possible pathophysiological mechanisms for SBI have been suggested (e.g., lipohyalinosis, atherosclerosis, and endothelial dysfunction), [4–6] however, there is still a lack of knowledge in this area.

For stroke clinicians, serum cholesterol level is a well-known risk factor. International guidelines recommend the control of lipid profiles, particularly low-density lipoprotein (LDL) levels [7, 8]. Recently, investigators have paid attention to the role of LDL particle phenotypes, rather than their total amounts, in the development of vascular complications [9, 10]. Small/dense LDL particles have atherogenic roles, while larger ones seem to have protective effects [11–14]. However, direct determination of the LDL phenotype is expensive and not yet standardized [9, 10, 15]. Thus, application in clinical practice is still premature.

The size of LDL particles is inversely determined by circulating triglyceride (TG) and very low-density lipoprotein (VLDL) levels [9, 10]. Using this nature, investigators can identify LDL phenotypes based on the ratio of TG and high-density lipoprotein (HDL), which has been confirmed in direct validation studies [9–12, 15, 16]. Similar to small/dense LDL particles, high TG/HDL cholesterol ratio is also associated with insulin resistance (IR), metabolic diseases, and cardio/cerebrovascular diseases [17–21]. In this study, we evaluated the relationship between TG/HDL cholesterol ratio and the prevalence of SBI in a neurologically healthy population.

## Methods

### Participants

**Fig. 1:**
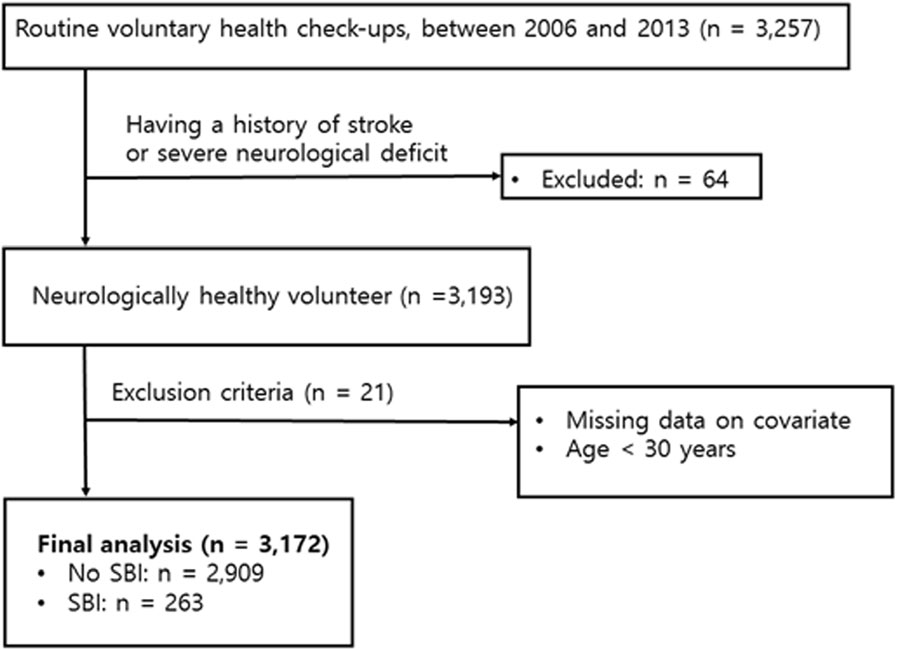
Patients inclusion flow-chart

We retrospectively evaluated 3257 participants from a consecutive registry of routine health check-ups at the Seoul National University Hospital Health Promotion Center between January 2006 and December 2013. Brain magnetic resonance imaging (MRI), magnetic resonance angiography (MRA), and laboratory examinations were conducted in all participants. Among them, 64 participants who had a history of stroke or severe neurological deficit were excluded. Subjects with missing covariate data (*n* = 18) and those under 30 years of age (*n* = 3) were also excluded. Finally, a total of 3172 neurologically healthy participants were analyzed (Fig. 1). The current study was approved by the Institutional Review Board at the Seoul National University Hospital (IRB number: H-1502-026-647) and any data not published within the article will be available from the corresponding author upon reasonable request.

### Clinical assessment

We evaluated demographic and metabolic factors, including age, sex, body mass index, hypertension, diabetes, hyperlipidemia, ischemic heart disease, current smoking, current alcohol use, and use of antiplatelet, antihypertensive or statin medication [22]. Laboratory examinations, including glucose profiles, lipid profiles, high-sensitivity C-reactive protein (hs-CRP) levels, and white blood cell counts, were also conducted after 12 h of overnight fasting [22].

Lipid profiles included those of total cholesterol (TC), LDL cholesterol, HDL cholesterol, and TG. The TG/HDL cholesterol ratio was calculated after dividing absolute TG levels by absolute HDL cholesterol levels in peripheral blood. Up to date, numerous novel indices have been developed from the classic lipid profiles, and they indicate additional information about LDL phenotype and vascular risk factors [8, 17, 21]. Thus, in addition to TG/HDL ratio, we also calculated TC/TG ratio which positively determines LDL particle size [8, 13].

### Radiological assessment

All participants underwent brain MRI and MRA using a 1.5-Tesla MR scanner (Magnetom SONATA, Siemens, Munich, Germany or Signa, GE Healthcare, Milwaukee, WI, USA). The basic thickness of brain MRI was 5 mm, and detailed acquisition parameters were as follows: T1-weighted images (repetition time [TR]/echo time [TE] = 500/11 ms); T2 fluid-attenuated inversion recovery images (TR/TE = 8800/127 ms); T2-weighted images (TR/TE = 5000/127 ms); T2-gradient echo images (TR/TE = 57/20 ms); and three-dimensional time-of-flight MRA images (TR/TE = 24/3.5 ms, slice thickness = 1.2 mm).

SBI was defined as an asymptomatic, well-defined lesion of ≥3 mm with the same signal characteristics as cerebrospinal fluid on T1- or T2-weighted images [1]. The burden of SBI lesions was classified as absent, single, or multiple. Intracranial atherosclerosis (ICAS) was defined as an occlusion or stenosis greater than 50% of an intracranial vessel on MRA [23]. All radiological markers were rated by two neurologists (K.-W.N. and H.-Y.J.). Disagreements were resolved by discussion with a third rater (H.-M.K.).

### Statistical analysis

All statistical analyses were performed using SPSS version 23 (IBM SPSS, Chicago, IL, USA). To compare the baseline characteristics between participants with SBI and those without SBI, Student’s *t*-test or the Mann-Whitney *U*-test were used for continuous variables, and the chi-squared test or Fisher’s exact test were used for categorical variables. Variables with *P* <  0.05 in the univariate analysis and sex, LDL cholesterol were introduced as confounders in the multivariate logistic regression analysis. To eliminate the effect of lipid-lowering agents, we conducted additional sensitivity analysis after excluding 233 participants who were taking statin. Lipid profiles, especially TG and HDL cholesterol levels, are highly influenced by sex [19, 24]. Therefore, we also conducted additional subgroup analyses in both male and female groups. Furthermore, to understand underlying pathophysiology, we compared various lipid parameter according to SBI lesion burden using the Kruskal-Wallis test, and the Jonckheere-Terpstra test. In this study, statistical significance was considered at *P* <  0.05.

## Results

A total of 3172 healthy subjects were assessed (median age: 56 years; male sex: 54.0%). SBI lesions were found in 263 (8.3%) participants, and the median TG/HDL cholesterol ratio was 1.87 [1.23–3.02]. The baseline characteristics of the cohort are presented in Table 1. In univariate analyses, SBI (+) group was associated with age, hypertension, diabetes, antiplatelet medication, ICAS, and levels of fasting glucose, TC, LDL cholesterol, HDL cholesterol, TG, non-HDL cholesterol, TG/HDL cholesterol ratio, TC/TG ratio, and hs-CRP (Table 2).

**Table 1:** Baseline characteristics of the cohort (*n* = 3172)

	Total	Male (*n* = 1713)	Female (*n* = 1459)
Age, y [IQR]	56 [50–63]	56 [50–63]	57 [51–63]
Sex, male, n (%)	1713 (54.0)	N/A	N/A
Body mass index, kg/m^2^ [IQR]	24.03 [22.11–25.96]	24.48 [22.73–26.35]	23.47 [21.53–25.43]
Hypertension, n (%)	712 (22.4)	420 (24.5)	292 (20.0)
Diabetes, n (%)	437 (13.8)	390 (16.9)	147 (10.1)
Hyperlipidemia, n (%)	808 (25.5)	403 (23.5)	405 (27.8)
Ischemic heart disease, n (%)	124 (3.9)	73 (4.3)	51 (3.5)
Current smoking, n (%)	489 (15.4)	445 (26.0)	44 (3.0)
Current alcohol use, n (%)	1539 (48.5)	1148 (67.0)	391 (26.8)
On antiplatelet medication, n (%)	326 (10.3)	210 (12.3)	116 (8.0)
On antihypertensive, n (%)	699 (22.0)	371 (21.7)	328 (22.5)
On statin, n (%)	256 (8.1)	143 (8.3)	113 (7.7)
Fasting glucose, mg/dL [IQR]	91 [85–101]	93 [86–104]	90 [84–99]
Total cholesterol, mg/dL [IQR]	198 [174–223]	194 [172–219]	202 [178–227]
LDL cholesterol, mg/dL [IQR]	124 [101–147]	123 [101–147]	126 [102–148]
HDL cholesterol, mg/dL [IQR]	53 [45–63]	49 [42–58]	57 [49–67]
Triglycerides, mg/dL [IQR]	100 [73–144]	110 [78–159]	91 [69–125]
TG/HDL cholesterol ratio [IQR]	1.87 [1.23–3.02]	2.21 [1.43–3.56]	1.56 [1.09–2.42]
TC/TG ratio [IQR]	1.95 [1.36–2.67]	1.74 [1.23–2.44]	2.21 [1.60–2.94]
White blood cells, × 10^3^/μL [IQR]	5.31 [4.40–6.37]	5.61 [4.66–6.82]	5.03 [4.15–5.98]
hs-CRP, mg/dL [IQR]	0.04 [0.01–0.15]	0.06 [0.01–0.16]	0.03 [0.01–0.13]
Silent brain infarct, n (%)	263 (8.3)	146 (8.5)	117 (8.0)
Intracranial atherosclerosis, n (%)	95 (3.0)	51 (3.0)	44 (3.0)

*LDL* low-density lipoprotein, *HDL* high-density lipoprotein, *TG* triglyceride, *TC* total cholesterol, *hs-CRP* high-sensitivity C-reactive protein

**Table 2:** Differences of characteristics between patients with and without SBI

	No SBI (*n* = 2909)	SBI (*n* = 263)	*P*-value
Age, y [IQR]	56 [50–62]	63 [57–69]	< 0.001
Sex, male, n (%)	1567 (53.9)	146 (55.5)	0.608
Body mass index, kg/m^2^ [IQR]	24.00 [22.12–25.95]	24.24 [22.05–26.18]	0.357
Hypertension, n (%)	619 (21.3)	93 (35.4)	< 0.001
Diabetes, n (%)	378 (13.0)	59 (22.4)	< 0.001
Hyperlipidemia, n (%)	737 (25.4)	71 (27.0)	0.562
Ischemic heart disease, n (%)	110 (3.8)	14 (5.3)	0.217
Current smoking, n (%)	455 (15.6)	34 (12.9)	0.243
Current alcohol use, n (%)	1419 (48.8)	120 (45.6)	0.327
On antiplatelet medication, n (%)	285 (9.8)	41 (15.6)	0.003
On antihypertensive, n (%)	635 (21.8)	64 (24.3)	0.348
On statin, n (%)	233 (8.0)	23 (8.7)	0.675
Fasting glucose, mg/dL [IQR]	91 [85–101]	94 [85–109]	0.003
Total cholesterol, mg/dL [IQR]	198 [175–223]	191 [166–218]	0.003
LDL cholesterol, mg/dL [IQR]	125 [102–147]	117 [90–147]	0.009
HDL cholesterol, mg/dL [IQR]	53 [45–63]	51 [43–61]	0.028
Triglycerides, mg/dL [IQR]	99 [72–144]	107 [77–148]	0.037
TG/HDL cholesterol ratio [IQR]	1.85 [1.22–2.98]	2.12 [1.31–3.38]	0.016
TC/TG ratio [IQR]	1.97 [1.39–2.69]	1.74 [1.20–2.49]	0.002
White blood cells, × 10^3^/μL [IQR]	5.30 [4.40–6.36]	5.48 [4.44–6.77]	0.054
hs-CRP, mg/dL [IQR]	0.04 [0.01–0.15]	0.07 [0.01–0.17]	0.039
Intracranial atherosclerosis, n (%)	80 (2.8)	15 (5.7)	0.007

*LDL* low-density lipoprotein, *HDL* high-density lipoprotein, *TG* triglyceride, *TC* total cholesterol, *hs-CRP* high-sensitivity C-reactive protein

In multivariate analysis to evaluate the possible predictors of SBI, TG/HDL cholesterol ratio (adjusted odds ratio [aOR] = 1.16, 95% confidence interval [CI] = 1.00 to 1.34, *P* = 0.047) remained a significant predictor of SBI, after adjustment for confounders (Table 3). Age and hypertension were also associated with SBI, independently of TG/HDL cholesterol ratio. Furthermore, this association was more prominent in males (aOR = 1.23, 95% CI = 1.03 to 1.48, *P* = 0.021), but not in females. When we conducted additional sensitivity analysis to exclude the effect of statins, the close association between TG/HDL cholesterol ratio and SBI was confirmed (Additional file 1).

**Table 3:** Multivariate analysis of possible predictors of silent brain infarct

	Crude OR (95% CI)	*P-*value	Adjusted OR (95% CI)	*P-*value
Total				
Age^a^	2.28 (1.98 to 2.63)	< 0.001	1.10 (1.09 to 1.12)	< 0.001
Sex	1.07 (0.83 to 1.38)	0.608	1.02 (0.75 to 1.39)	0.890
Hypertension	2.02 (1.55 to 2.65)	< 0.001	1.17 (0.83 to 1.65)	0.037
Diabetes	1.94 (1.42 to 2.64)	< 0.001	1.31 (0.90 to 1.89)	0.157
On antiplatelet medication	1.70 (1.19 to 2.43)	0.003	1.20 (0.80 to 1.79)	0.372
hs-CRP^a^	1.12 (1.03 to 1.21)	0.008	1.07 (0.96 to 1.18)	0.219
ICAS	2.14 (1.21 to 3.77)	0.009	0.94 (0.44 to 2.00)	0.865
LDL cholesterol^a^	0.83 (0.71 to 0.96)	0.012	0.96 (0.83 to 1.13)	0.637
TG/HDL cholesterol ratio^a^	1.13 (1.01 to 1.26)	0.030	1.16 (1.00 to 1.34)	0.047
Male				
Age^a^	2.44 (2.02 to 2.94)	< 0.001	1.11 (1.08 to 1.14)	< 0.001
Hypertension	2.27 (1.60 to 3.22)	< 0.001	1.44 (0.92 to 2.24)	0.111
Diabetes	1.69 (1.13 to 2.52)	0.010	1.14 (0.71 to 1.85)	0.587
On antiplatelet medication	1.99 (1.30 to 3.07)	0.002	1.31 (0.79 to 2.16)	0.294
hs-CRP^a^	1.17 (1.06 to 1.29)	0.002	1.08 (0.96 to 1.22)	0.206
ICAS	2.06 (0.95 to 4.46)	0.068	1.28 (0.49 to 3.32)	0.617
LDL cholesterol^a^	0.74 (0.60 to 0.90)	0.003	0.94 (0.76 to 1.17)	0.590
TG/HDL cholesterol ratio^a^	1.08 (0.94 to 1.24)	0.289	1.23 (1.03 to 1.48)	0.021
Female				
Age^a^	2.09 (1.69 to 2.59)	< 0.001	1.09 (1.06 to 1.12)	< 0.001
Hypertension	1.72 (1.13 to 2.62)	0.012	0.88 (0.51 to 1.54)	0.656
Diabetes	2.40 (1.47 to 3.93)	< 0.001	1.72 (0.95 to 3.11)	0.073
On antiplatelet medication	1.22 (0.64 to 2.35)	0.546	0.92 (0.45 to 1.86)	0.811
hs-CRP^a^	1.01 (0.84 to 1.23)	0.898	1.02 (0.82 to 1.27)	0.848
ICAS	2.24 (0.98 to 5.15)	0.057	0.67 (0.19 to 2.39)	0.532
LDL cholesterol^a^	0.95 (0.76 to 1.19)	0.657	1.00 (0.80 to 1.26)	0.989
TG/HDL cholesterol ratio^a^	1.26 (1.03 to 1.53)	0.023	1.06 (0.82 to 1.36)	0.672

*hs-CRP* high-sensitivity C-reactive protein, *ICAS* intracranial atherosclerosis, *TG* triglyceride, *HDL* high-density lipoprotein

^a^These variables were standardized by division by the standard deviation

**Fig. 2:**
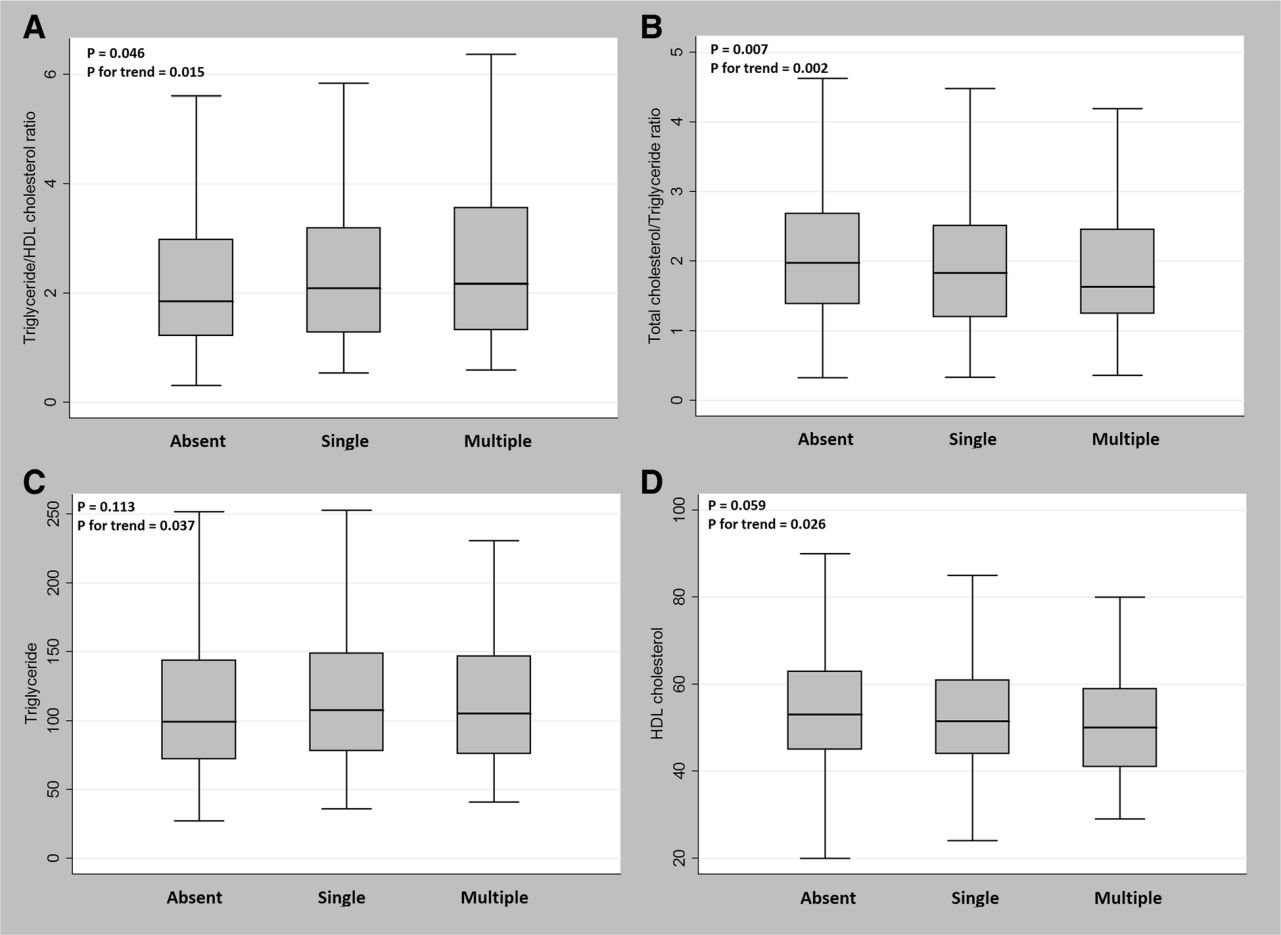
The association between lipid parameters and SBI lesion burden. **a** TG/HDL cholesterol ratio correlated positively and **b** TC/TG ratio correlated negatively with SBI lesion burden, in dose-response manners (P for trend = 0.015 and 0.002, respectively). **c** TG and **d** HDL cholesterol also showed tendencies toward correlations with SBI lesion burden in dose-response manners, yet not significantly so

In the analyses of the relationships between lipid parameters and SBI lesion burden, TG/HDL cholesterol ratio was positively correlated (*P* = 0.046) and TC/TG ratio was negatively correlated (*P* = 0.007) with SBI lesion burden, even in dose-response manners (*P* for trend = 0.015 and 0.002, respectively) (Fig. 2). There was no significant correlation between TG (*P* = 0.113) or HDL cholesterol (*P* = 0.059) and SBI lesion burden; however, they presented tendencies for dose-response relationships (*P* for trend = 0.037 and 0.026, respectively).

## Discussion

In this study, we demonstrated that TG/HDL cholesterol ratio was positively associated with the prevalence of SBI in a neurologically healthy population. Since this association also occurred in a dose-response manner, our findings may suggest clues for the underlying pathophysiologic mechanisms.

Obviously, our main findings revealed that a pattern of “high-TG and low-HDL cholesterol” pattern may be harmful. This “atherogenic dyslipidemia” has been recently focused, because it is considered as a surrogate marker of small/dense LDL particles and IR status [11, 19, 25, 26]. Our results could be interpreted in two ways: first, as we mentioned, high TG/HDL cholesterol ratio indicates harmful small/dense LDL particles that could contribute to cerebrovascular diseases [12–14, 25]. Another index (i.e., TC/TG ratio), which positively reflects LDL particle size, [13] also confirmed this idea. Second, the hypertriglyceridemia itself could have harmful effects on cerebrovascular diseases, which is in line with previous studies [27, 28]. Regardless of the interpretation, high TG levels, which are treated with a little different medication from that for high TC or LDL levels, should be controlled.

The exact mechanisms underlying the relationship between TG/HDL cholesterol ratio and SBI are unclear. However, we suggest several plausible explanations: first, high TG/HDL cholesterol ratio may indicate higher atherosclerosis burden. TG/HDL cholesterol ratio is closely associated with atherosclerosis regardless of their stenosis degree [8, 17, 29, 30]. It can result from small/dense LDL particles which are susceptible to oxidation, leading to atherogenesis [12, 26]. VLDL, which coexists with atherogenic dyslipidemia, also accelerates atherosclerosis by being taken into macrophages and developing foam cells [26]. Because advanced atherosclerosis can result in diffuse-hypoperfusion, extravasation of toxic metabolites into neural tissues, and occlusion of small arterioles, [22] high TG/HDL cholesterol may ratio be associated with the prevalence of SBI through higher atherosclerosis burden; second, inflammation and oxidative stress may play a role. TG/HDL cholesterol ratio indicates IR status, [17, 18, 29, 31, 32] and then, it also means increased subclinical inflammation, disturbed metabolic status, and elevated sympathetic tone [31, 33]. This high inflammation burden promote downstream of lipid peroxidation and cellular/DNA damage, leading to endothelial dysfunction/arterial stiffness. Supporting these ideas, several studies have reported a direct relationship between TG/HDL cholesterol ratio and arterial stiffness. We also found that subjects with higher TG/HDL cholesterol ratio had higher levels of inflammatory markers (e.g., hs-CRP and white blood cell counts) (Table 4) [14, 17]. Because endothelial dysfunction/arterial stiffness is one of leading causes of SBI development, [5] subclinical inflammation and endothelial dysfunction may provide a connection between TG/HDL cholesterol ratio and SBI prevalence; lastly, TG/HDL cholesterol ratio could be a simple surrogate marker of subjects who have numerous vascular risk factors. We already knew that TG/HDL cholesterol ratio is related to various metabolic risk factors that are also risk factors for SBI [17–19]. Thus, subjects with higher TG/HDL cholesterol ratio may have additional vascular risk factors that contribute to SBI (Table 4).

**Table 4:** Comparisons of risk factors according to TG/HDL cholesterol ratio teritles

	Tertile 1 (< 1.42)	Tertile 2 (1.42–2.56)	Tertile 3 (> 2.56)	*P* for trend
Number	1057	1056	1059	
Age, y [IQR]	55 [50–63]	57 [52–63]	56 [49–63]	0.386
Sex, male, n (%)	421 (39.7)	555 (52.7)	737 (69.7)	< 0.001
Body mass index, kg/m^2^ [IQR]	22.92 [21.11–24.80]	24.14 [22.26–26.00]	24.98 [23.37–26.93]	< 0.001
Hypertension, n (%)	184 (17.3)	255 (24.2)	273 (25.8)	< 0.001
Diabetes, n (%)	99 (9.3)	143 (13.6)	195 (18.4)	< 0.001
Ischemic heart disease, n (%)	40 (3.8)	36 (3.4)	48 (4.5)	0.361
Current smoking, n (%)	86 (8.1)	124 (11.8)	279 (26.4)	< 0.001
Current alcohol use, n (%)	467 (44.0)	505 (47.9)	567 (53.6)	< 0.001
Fasting glucose, mg/dL [IQR]	89 [82–97]	92 [85–101]	95 [87–105]	< 0.001
White blood cells, × 10^3^/μL [IQR]	4.81 [4.00–5.83]	5.28 [4.42–6.21]	5.90 [4.92–7.12]	< 0.001
hs-CRP, mg/dL [IQR]	0.02 [0.01–0.11]	0.05 [0.01–0.15]	0.09 [0.01–0.18]	< 0.001

*hs-CRP* high-sensitivity C-reactive protein

Interestingly, our results were more prominent in male participants. The exact reason for this sexual difference is unclear. However, we suggest several possible explanations: first, males had higher TG and lower HDL cholesterol values than those of females. Thus, the ratio of participants who had abnormal TG/HDL cholesterol ratio to normal ones may be prominent in males, and males had larger effect size. This could make more prominent association between TG/HDL cholesterol ratio and SBI. second, as we shown in Table 1, male participants had more frequent vascular risk factors than females, except for older age, and all of these metabolic and inflammatory factors were also closely related to TG/HDL cholesterol ratio (Table 4). TG/HDL cholesterol ratio in male group might more clearly reflect vulnerable conditions underlying SBI development than female group. Last, another explanation is based on the distribution of the adipose tissue according to sex. Males have higher proportions of visceral adipose tissues, which are the sources of circulating TG, while females have a lot of subcutaneous adipose tissues with metabolically protective effects [34]. These biological discrepancies may explain the difference in TG/HDL cholesterol ratio, and its effect on SBI development.

Our study has several considerable limitations. First, it was designed as a retrospective, single-center study. We included a relatively large number of participants, and broadly evaluated their lipid profiles and brain MRI data. However, the possibility of selection bias still exists. Second, due to the nature of the cross-sectional analyses, we could not establish a causal relationship between TG/HDL cholesterol ratio and SBI. Further prospective randomized controlled studies are needed to identify causal relationships and underlying patho-mechanisms. Third, we could not directly measure small/dense LDL particle levels. If we included directly measured small/dense LDL particle levels, we could investigate whether TG/HDL cholesterol ratio is only a surrogate marker for small/dense LDL particles or it also plays an individual role in SBI development. Fourth, although we have considered the effects of statin on our outcomes, other lipid-lowering agents such as fenofibrate may also influence the relationship between SBI and TG/HDL ratio. Last, SBI can not only be from atherosclerotic vascular diseases. Bias from other possible pathologies (e.g., vasculitis, dissection, cardioembolic sources, hypercoagulability) should be considered.

## Conclusion

A high TG/HDL cholesterol ratio may be a risk factor for the prevalence and burden of SBI lesions in a neurologically healthy population. Because SBI is a risk factor for subsequent ischemic stroke, [2] our simple, inexpensive, but efficient method could identify modifiable high-risk group which requires early intervention. However, our findings should be confirmed with further prospective studies.

## Additional file

Additional file 1: Multivariate analysis of possible predictors of silent brain infarct, excluding patients with statin use† (*n* = 2916). (DOCX 15 kb)

## Data Availability

The datasets during and/or analyzed during the current study available from the corresponding author on reasonable request.

## Abbreviations

HDL: High-density lipoprotein
hs-CRP: High-sensitivity C-reactive protein
ICAS: Intracranial atherosclerosis
IR: Insulin resistance
LDL: Low-density lipoprotein
SBI: Silent brain infarct
TC: Total cholesterol
TE: Echo time
TG: Triglyceride
TR: Repetition time
VLDL: Very low-density lipoprotein
