# Supplementary material for: High triglyceride/HDL cholesterol ratio is associated with silent brain infarcts in a healthy population
Source: BMC Neurol. 2019 Jul 2;19:147. doi: 10.1186/s12883-019-1373-8 (PMC6604433; doi:10.1186/s12883-019-1373-8)
Supplement: Supplementary file 1 — Multivariate analysis of possible predictors of silent brain infarct, excluding patients with statin use† (n = 2916). (DOCX 15 kb) [file 12883_2019_1373_MOESM1_ESM.docx]

**Additional file 1. Multivariate analysis of possible predictors of silent brain infarct, excluding patients with statin use**^†^ **(n = 2,916)**

|  | **Crude OR (95% CI)** | ***P-*value** | **Adjusted OR (95% CI)** | ***P-*value** |
| --- | --- | --- | --- | --- |
| Age^*^ | 2.17 (1.87 to 2.51) | < 0.001 | 1.10 (1.07 to 1.12) | < 0.001 |
| Sex | 1.11 (0.85 to 1.45) | 0.435 | 1.05 (0.76 to 1.46) | 0.762 |
| Hypertension | 2.11 (1.60 to 2.79) | < 0.001 | 1.29 (0.91 to 1.85) | 0.155 |
| Diabetes | 1.90 (1.37 to 2.63) | < 0.001 | 1.38 (0.94 to 2.03) | 0.102 |
| On antiplatelet medication | 1.65 (1.13 to 2.40) | 0.009 | 1.15 (0.75 to 1.76) | 0.519 |
| hs-CRP^*^ | 1.12 (1.03 to 1.21) | 0.010 | 1.12 (0.52 to 2.40) | 0.781 |
| ICAS | 2.48 (1.40 to 4.41) | 0.002 | 1.06 (0.96 to 1.17) | 0.268 |
| LDL cholesterol^*^ | 0.85 (0.73 to 0.99) | 0.041 | 0.98 (0.83 to 1.15) | 0.787 |
| TG/HDL cholesterol ratio^*^ | 1.11 (0.99 to 1.25) | 0.078 | 1.17 (1.00 to 1.38) | 0.049 |

hs-CRP = high-sensitivity C-reactive protein, ICAS = intracranial atherosclerosis, LDL = low-density lipoprotein, TG = triglyceride, HDL = high-density lipoprotein

^*^These variables were standardized by division by the standard deviation.

^†^These analyses were performed after excluding subjects with statin medication
